# Morpho-phylogenetic evidence reveals new species in Rhytismataceae (Rhytismatales, Leotiomycetes, Ascomycota) from Guizhou Province, China

**DOI:** 10.3897/mycokeys.76.58465

**Published:** 2020-12-31

**Authors:** Jin-Feng Zhang, Jian-Kui Liu, Kevin D. Hyde, Anusha H. Ekanayaka, Zuo-Yi Liu

**Affiliations:** 1 Institute of Tea Research, Guizhou Academy of Agricultural Sciences, Guiyang 550006, China Guizhou Key Laboratory of Agriculture Biotechnology, Guizhou Academy of Agricultural Sciences Guiyang China; 2 Guizhou Key Laboratory of Agriculture Biotechnology, Guizhou Academy of Agricultural Sciences, Guiyang 550006, China nstitute of Tea Research, Guizhou Academy of Agricultural Sciences Guiyang China; 3 Center of Excellence in Fungal Research and School of Science, Mae Fah Luang University, Chiang Rai 57100, Thailand Mae Fah Luang University Muang Thailand; 4 School of Life Science and Technology, University of Electronic Science and Technology of China, Chengdu 611731, China University of Electronic Science and Technology of China Chengdu China; 5 Innovative Institute of Plant Health, Zhongkai University of Agriculture and Engineering, Haizhu District, Guangzhou 510225, China Zhongkai University of Agriculture and Engineering Guangzhou China; 6 Key Laboratory for Plant Diversity and Biogeography of East Asia, Kunming Institute of Botany, Chinese Academy of Sciences, Kunming 650201, China Kunming Institute of Botany, Chinese Academy of Sciences Kunming China

**Keywords:** four new taxa, *
Hypoderma
*, karst formations, taxonomy, *
Terriera
*

## Abstract

Karst formations represent a unique eco-environment. Research in the microfungi inhabiting this area is limited. During an ongoing survey of ascomycetous microfungi from karst terrains in Guizhou Province, China, we discovered four new species, which are introduced here as *Hypoderma paralinderae*, *Terriera karsti*, *T. meitanensis* and *T. sigmoideospora* placed in Rhytismataceae, based on phylogenetic analyses and morphological characters. Molecular analyses, based on concatenated LSU-ITS-mtSSU sequence data, were used to infer phylogenetic affinities. Detail descriptions and comprehensive illustrations of these new taxa are provided and relationships with the allied species are discussed, based on comparative morphology and molecular data.

## Introduction

Rhytismataceae (Rhytismatales) was established by Chevallier (1826), typified by *Rhytisma* with *R. acerinum* (Pers.) Fr. as the type species and belongs in Rhytismatales, Leotiomycetes, Ascomycota (Wijayawardene et al. 2020). Members of this family produce variously shaped apothecia that may be sessile, circular, navicular or hysteriform and that typically open by a longitudinal split or radial fissures. Asci are cylindrical, saccate to clavate. Ascospores are one-celled or multi-septate and vary from bacilliform to fusiform or filiform, with or without a sheath (Darker 1967; Ekanayaka et al. 2019). Species of Rhytismataceae occur on a wide range of hosts with a worldwide distribution (Cannon and Minter 1986; Johnston 1986; Hou and Piepenbring 2009; Hernández et al. 2014; Li et al. 2014; Tanney and Seifert 2017; Cai et al. 2020).

Darker (1967) proposed the generic delimitation for Rhytismataceae, based on ascoma and ascospore shapes, although this has been challenged in later studies (Cannon and Minter 1986; Johnston 1990, 2001; Hou et al. 2005). However, Darker (1967) and Cannon and Minter (1986) were followed due to lack of an alternative scheme. Molecular studies (Gernandt et al. 2001; Johnston and Park 2007; Lantz et al. 2011; Tian et al. 2013; Zhang et al. 2015) had revealed the phylogenetic relationships amongst members of Rhytismatales, but the available sequence data for this group remains limited and a phylogenetic classification of some members is unresolved. There are around 50 genera with 1000 species presently accepted in Rhytismataceae (Lumbsch and Huhndorf 2007; Wijayawardene et al. 2018; Index Fungorum 2020); however, a systematic genus-level taxonomic revision is needed to provide a clear, natural generic delimitation within this family and the relationship between Rhytismataceae and allied families within Rhytismatales needs to be resolved (Johnston et al. 2019).

Karst formations are generally characterised by sinking streams, caves, enclosed depressions, fluted rock outcrops and large springs (Ford and Williams 2007). Guizhou, as the eastern portion of the Yunnan-Guizhou Plateau, has the largest proportion of rocky desertification and karst landforms in China (Huang and Cai 2006). The flora in this area, comprising of 264 families with 1667 genera and 7505 vascular plants species, were inventoried from Guizhou Province (Liu et al. 2018). Therefore, it would be interesting to study the fungi in this area because of its unique ecological environment and rich plant resources. A series of studies have already been carried out and yielded several new species (Zhang et al. 2016, 2017a, b, 2018, 2019). The objectives of this study are to introduce four novel species of Rhytismataceae, based on phylogenetic and morphological evidence and elucidate their affinities with related species.

## Materials and methods

### Collection, examination, isolation and specimen deposition

Specimens were collected from Guizhou Province from 2016 to 2017 and examined in the laboratory with a Motic SMZ 168 stereomicroscope. Vertical sections of fruiting bodies were made by hand and mounted in water for microscopy. Macro-morphological characters were captured using a stereomicroscope (Nikon SMZ800N) with a Cannon EOS 70D digital camera. Micro-morphological characters were observed by differential interference contrast (DIC) using a Nikon ECLIPSE 80*i* compound microscope and captured by a Cannon EOS 600D digital camera. Measurements were processed in a Tarosoft (R) Image Frame Work version 0.9.7 programme and photographic plates were edited in Adobe Photoshop CS6 (Adobe Systems Inc., USA).

The single spore isolation technique described in Chomnunti et al. (2014) was followed to obtain the pure cultures of these specimens. Single germinated ascospore was picked up and transferred to potato dextrose agar (PDA; 39 g/l distilled water, Difco potato dextrose) for recording growth rates and culture characteristics.

The holotypes are deposited at the Herbarium of Mae Fah Luang University (**MFLU**), Chiang Rai, Thailand or Guizhou Academy of Agricultural Sciences (**GZAAS**), Guizhou, China. Ex-type living culture is deposited at Guizhou Culture Collection (**GZCC**), Guiyang, China. Index Fungorum and Facesoffungi numbers are provided according to Jayasiri et al. (2015) and Index Fungorum (2020). New species were established, based on the recommendations from Jeewon and Hyde (2016).

### DNA extraction, PCR and phylogenetic analyses

Following the manufacturer’s instructions, the total genomic DNA was extracted from cultures using a Biospin Fungus Genomic DNA Extraction Kit (BioFlux, Hangzhou, P. R. China) or extracted from the fruiting bodies using an E.Z.N.A. Forensic DNA kit (Omega Bio-Tek, Doraville, Georgia, USA).

Polymerase chain reactions (PCR) were performed in 25 μl reaction volumes, which contained 9.5 μl distilled-deionised-water, 12.5 μl of 2 × Power Taq PCR Master Mix (TIANGEN Co., China), 1 μl of DNA template and 1 μl of each forward and reverse primers. Three different loci were used in this study. The internal transcribed spacer (ITS) and 28S large subunit of the nuclear ribosomal DNA (LSU) regions were amplified by using the primers ITS4/ITS5 and LR0R/LR5, respectively (White et al. 1990; Gardes and Bruns 1993). The primers mrSSU1 and mrSSU3R were used for amplification of the mitochondrial small subunit (mtSSU) partial regions (Zoller et al. 1999). The PCR thermal cycle programme was performed according to White et al. (1990), Gardes and Bruns (1993) and Zoller et al. (1999). Amplicon size and concentration were assessed by gel electrophoresis with 1.2% agarose stained with ethidium bromide. PCR products were purified and sequenced at Sangon Biotechnology Co. Ltd (Shanghai, P. R. China).

For phylogenetic reconstruction, newly-generated sequences were initially subjected to BLAST search (BLASTn) in NCBI (https://www.ncbi.nlm.nih.gov) and additional related sequences were selected and downloaded from GenBank (https://www.ncbi.nlm.nih.gov/genbank/), based on BLASTn results and recent publications (Tian et al. 2013; Wang et al. 2013; Zhang et al. 2015; Johnston et al. 2019; Cai et al. 2020). The sequences used in this study for phylogenetic analysis are listed in Table 1. All of these sequences were aligned and manually improved with BioEdit v. 7.2 (Hall 1999) and then assembled as a dataset of LSU-ITS-mtSSU to infer the phylogenetic placement of newly identified taxa.

**Table 1.** Taxa used in this study. Strains generated/sequenced in this study are given in bold.

Taxa	Specimen/Strain No.	GenBank accession numbers		
		LSU	ITS	mtSSU
*Bifusella camelliae*	HOU 1094	KF797447	KF797435	KF797458
	HOU 701B	KF797448	KF797436	KF797459
*Coccomyces anhuiensis*	BJTC 201610	MK371314	MK371313	MK371315
*Coccomyces dentatus*	AFTOL ID-147	AY544657	DQ491499	AY544736
*Colpoma ledi*	Lantz 379 (UPS)	HM140512	–	HM143788
*Colpoma quercinum*	Lantz 368 (UPS)	HM140513	–	HM143789
*Cryptomyces maximus*	Lantz and Minter 424 (UPS)	HM140514	–	HM143790
*Discocainia nervalis*	BITC 201405	KJ513473	KJ507206	–
*Duplicariella phyllodoces*	Lantz 389 (UPS)	HM140516	–	–
*Hypoderma berberidis*	HOU 892	JX232420	JX232414	KF813010
	HOU 942	JX232421	JX232415	KF813009
*Hypoderma campanulatum*	ICMP 17383	HM140517	–	HM143792
*Hypoderma carinatum*	ICMP 18322	HM140518	–	HM143793
*Hypoderma cordylines*	ICMP 17344	HM140521	JF683421	HM143796
	ICMP 17396	HM140520	–	HM143795
*Hypoderma hederae*	Lantz and Minter 421 (UPS)	HM140522	JF690770	HM143797
*Hypoderma liliense*	ICMP 18323	HM140523	MH921859	HM143798
	ICMP 18324	HM140524	–	HM143799
*Hypoderma minteri*	BJTC 201203	JX232418	JX232416	–
*Hypoderma obtectum*	ICMP 17365	HM140525	–	HM143800
***Hypoderma paralinderae***	**GZAAS 19-1769**	**MN638878**	**MN638873**	**MN638868**
*Hypoderma rubi*	Hanson 2006-451 (UPS)	HM140519	JF690769	HM143794
	ICMP 17339	HM140526	JF683419	HM143801
	ICMP 18325	HM140527	JF683418	HM143802
	Lantz 405 (UPS)	HM140530	JF690772	HM143805
*Hypoderma sticheri*	ICMP 17353	HM140529	MK039702	HM143804
*Hypohelion anhuiense*	BITC 201311	KF797443	KF797431	KF797455
*Hypohelion scirpinum*	Lantz 394 (UPS)	HM140531	–	HM143806
*Lirula macrospora*	Hou et al. 13 (BJTC)	HQ902159	HQ902152	–
*Lirula yunnanensis*	BJTC 2012	HQ902149	HQ902156	–
*Lophodermium arundinaceum*	Lantz 323 (UPS)	HM140535	–	HM143811
*Lophodermium culmigenum*	ICMP 18328	HM140538	–	HM143814
*Marthamyces emarginatus*	ICMP 22854	MK599203	MH921869	MK598751
*Meloderma dracophylli*	ICMP 17343	HM140561	MH921871	HM143833
*Nematococcomyces oberwinkleri*	BJTC 201205	KC312686	–	KC312689
*Nematococcomyces rhododendri*	HOU 469A	KC312687	KU213975	KC312691
*Rhytisma huangshanense*	HOU 564	FJ495192	GQ253101	–
*Rhytisma salicinum*	Lantz 370 (UPS)	HM140566	–	–
*Sporomega degenerans*	Lantz 367 (UPS)	HM140567	–	HM143839
*Terriera camelliicola*	AAUF 66555	KP878552	–	KP878553
*Terriera cladophila*	Lantz & Minter 423 (UPS)	HM140568	–	HM143840
*Terriera elliptica*	BJTC 201419	KP878550	KP878549	KP878551
*Terriera guihzouensis*	BITC 2020149	MT549890	MT534526	–
	BITC 2020147	–	MT534519	MT549863
	BITC 2020148	–	MT534527	MT549874
	BITC 2020149	MT549872	MT534528	MT549865
	BITC 2020150	–	MT534591	MT549888
*Terriera houjiazhuangensis*	BITC 2020145	MT549889	MT549882	–
	BITC 2020146	MT549864	MT549879	MT549884
	BITC 2020192	MT549869	MT549883	–
*Terriera ilicis*	BJTC 2020141	MT549885	MT549875	MT549868
	BJTC 2020193	MT549873	MT549861	MT549886
	BJTC 2020142	MT549881	MT549877	MT549870
***Terriera karsti***	**MFLU 18-2288**	**MN638881**	**MN638876**	**MN638871**
***Terriera meitanensis***	**MFLU 18-2299**	**MN638879**	**MN638874**	**MN638869**
***Terriera meitanensis***	**MFLU 18-2301**	**MN638880**	**MN638875**	**MN638870**
*Terriera minor*	ICMP 13973	HM140570	–	HM143842
*Terriera pandanicola*	MFLU 16-1931	MH260320	MH275086	MW334971
***Terriera sigmoideospora***	**MFLU 18-2297**	**MN638882**	**MN638877**	**MN638872**
*Terriera thailandica*	MFLUCC 14-0818	KX765301	–	–
*Therrya abieticola*	HOU 447A	KP322580	KP322574	KP322587
*Tryblidiopsis pinastri*	CBS 445.71	MH871979	JF793678	AF431963
*Tryblidiopsis sichuanensis*	BJTC 201211	KC312683	KC312676	KC312692
*Tryblidiopsis sinensis*	BJTC 201212	KC312681	KC312674	KC312694

Phylogenetic analyses were performed using the algorithm of Maximum-Parsimony (MP) and Bayesian Inference (BI). MP analyses were run using PAUP v. 4.0b10 (Swofford 2002) with 1000 replications and inferred using the heuristic search option with 1000 random taxa. All characters were unordered and of equal weight and gaps were treated as missing data. Maxtrees was set as 1000, zero-length branches were collapsed and all equally parsimonious trees were saved. Clade stability was accessed using a bootstrap (BT) analysis with 1000 replicates, each with ten replicates of random stepwise addition of taxa (Hillis and Bull 1993).

BI analyses were carried out by using MrBayes v. 3.2 (Ronquist et al. 2012). The best-fit model (GTR+I+G for LSU, ITS and mtSSU) of evolution was estimated in MrModeltest 2.3 (Nylander 2008). Posterior Probabilities (PP) (Rannala and Yang 1996; Zhaxybayeva and Gogarten 2002) were determined by Markov Chain Monte Carlo sampling (MCMC) in MrBayes v. 3.2. Six simultaneous Markov chains were run for 10,000,000 generations and trees were sampled every 100^th^ generation. The temperature values were lowered to 0.15, burn-in was set to 0.25 and the run was automatically stopped as soon as the average standard deviation of split frequencies reached below 0.01.

The phylogram was visualised in TreeView (Page 1996) and edited in Adobe Illustrator CS v. 5 (Adobe Systems Inc., USA). The finalised alignment and tree were deposited in TreeBASE, submission ID: 27401 (http://www.treebase.org).

## Results

### Phylogenetic analyses

**Figure 1.**
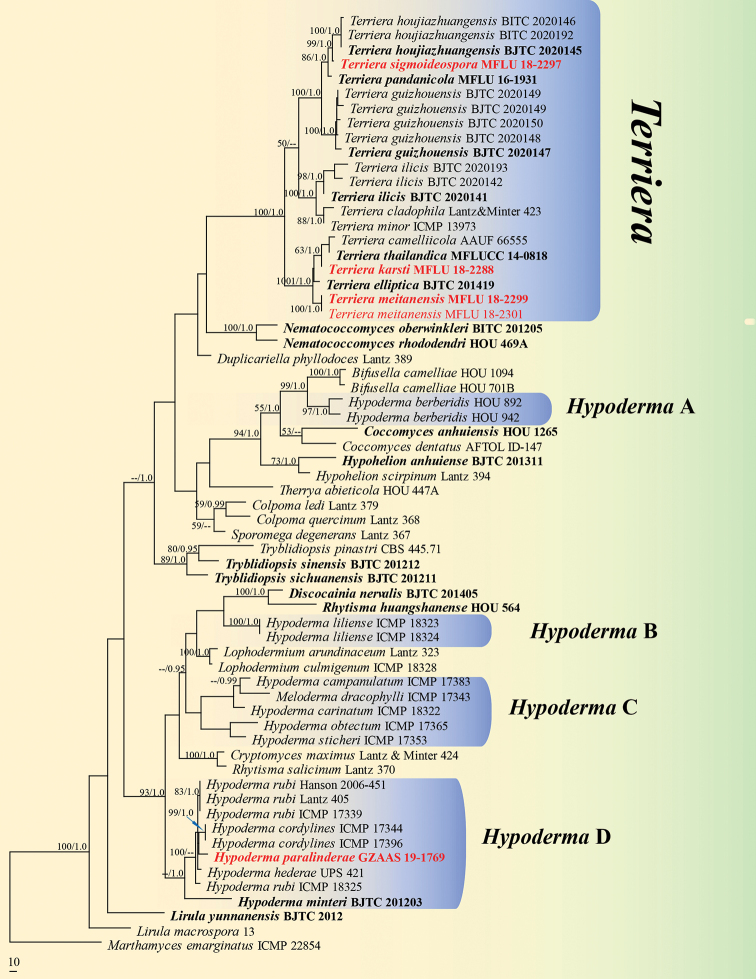
Phylogram of Rhytismataceae is presented as the best tree revealed by MP analysis, based on the concatenated LSU-ITS-mtSSU sequence dataset. MP bootstrap support values (MPBP ≥ 50%) and Bayesian inference posterior probabilities (BYPP ≥ 0.95) are shown near the nodes. The tree is rooted to *Marthamyces emarginatus* (ICMP 22854), the scale bar showing 10 changes. Type strains are indicated in bold and new sequences, generated in this study, are given in red.

The dataset for phylogenetic analysis comprised 64 strains, with *Marthamyces emarginatus* (Cooke & Massee) Minter selected as the outgroup taxon. This dataset consists of 2078 characters (including the gaps), of which 1205 are constant, 236 are variable parsimony-uninformative, while 637 characters are parsimony-informative. The most parsimonious tree showed with length of 2843 steps (CI = 0.480, RI = 0.759, RC = 0.364 and HI = 0.520). The best tree revealed by the MP analysis was selected to represent relationships amongst taxa (Fig. 1). The tree generated from Bayesian inference analyses had similar topology. The phylogram (Fig. 1) shows that *Hypoderma* is non-monophyletic (Clade A, B, C and D), with *H. paralinderae* clusters with three existing species viz. *H. cordylines* P.R. Johnst., *H. hederae* (T. Nees ex Mart.) De Not. and *H. rubi* (Pers.) DC. In contrast, all of the *Terriera* species with available sequences (including the newly generated sequences) form a monophyletic clade with strong statistical support (MPBP 100% and BYPP 1.00). This corresponds to the phylogeny in Zhang et al. (2015). *Terriera meitanensis* and *T. karsti* group together with three reported species viz. *T. camelliicola* (Minter) Y.R. Lin & C.L. Hou, *T. elliptica* T.T. Zhang & C.L. Hou and *T. thailandica* Jayasiri & K.D. Hyde, while *T. sigmoideospora* is placed within another clade that comprises *T. houjiazhuangensis* C.L. Hou & S.R. Cai and *T. pandanicola* Tibpromma & K.D. Hyde.

## Taxonomy

### *Hypoderma* De Not., G. bot. ital. 2(2): 13 (1847)

De Candolle (1805) introduced *Hypoderma* to accommodate taxa resembling *Hysterium* Pers., but with apothecia that are immersed in host-plant tissue and the hymenia are exposed via a longitudinal split in the substratum. Subsequently, the nomenclature of *Hypoderma* was challenged by various authors (Chevallier 1822, 1826; Fries 1823; Wallroth 1833). De Notaris (1847) recognised the distinction between *Hypoderma* and *Lophodermium* Chevall. and separated them, based on the ascospore shapes. So far, there are 214 epithets included in Index Fungorum (2020), but around half of these species are synonymized under other genera, such as *Lophodermium*, *Meloderma* Darker and *Terriera*.

#### Hypoderma paralinderae

Taxon classificationFungiRhytismatalesRhytismataceae

J.F. Zhang & Z.Y. Liu sp. nov.

AF0676BC-593E-5D83-83F0-5D6452E92C3D

Index Fungorum number: IF556909

Facesoffungi Number No: FoF06797

Figure 2

##### Etymology.

Referring to the morphological similarity with *Hypoderma linderae*.

##### Holotype.

GZAAS 19-1769.

##### Description.

**Figure 2.**
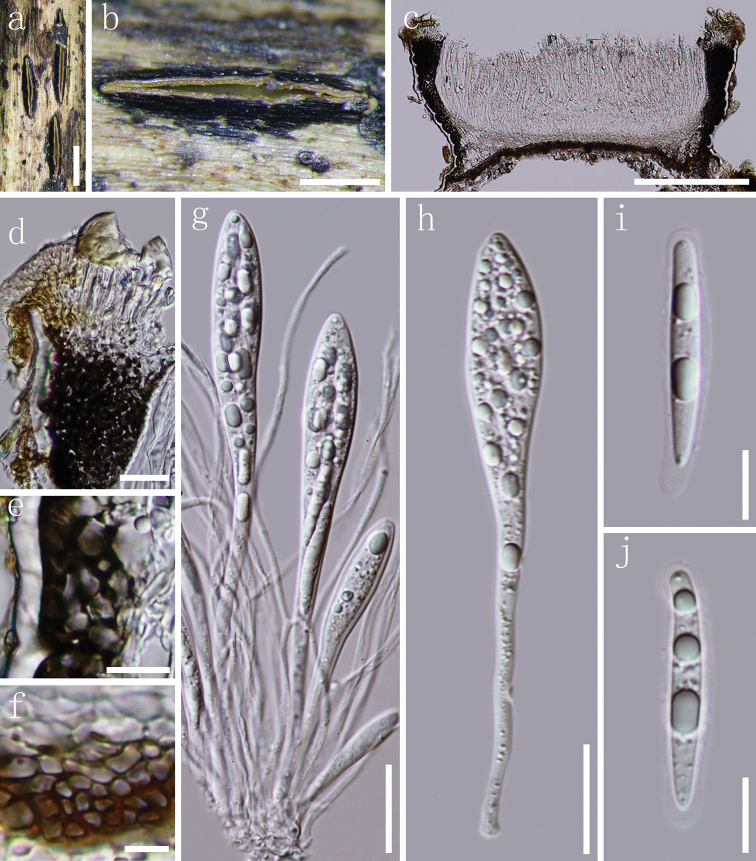
*Hypoderma paralinderae***a, b** apothecia observed under a dissecting microscope in face view **c** vertical section through an apothecium **d** lips adjacent to the top of covering stroma **e** section of covering stroma **f** section of basal stroma **g** paraphyses and asci in various states of maturity **h** immature ascus **i, j** ascospores. Note: **c–j** mounted in water. Scale bar: 1 mm (**a**), 500 µm (**b**), 200 µm (**c**), 20 µm (**d, g, h**), 10 µm (**e, i, j**), 5 µm (**f**).

*Apothecia* developing on dead stems, scattered, dark brown to black, shiny, long elliptical to slightly fusiform, straight or somewhat curved, ends rounded or obtuse, rising above the surface of the substrate, opening by a single longitudinal split. *Lips* moderately developed, pale brown (Fig. 2a, b). In median vertical section (Fig. 2c), apothecia subcuticular, 200–280 µm deep. *Covering stroma* (Fig. 2e) up to 38–45 µm thick near the opening, becoming to 12–18 µm thick towards the edges, extending to the basal stroma, consisting of an outer layer of host cuticle and several layers of dark brown, thick-walled cells of *textura angularis*. *Lip cells* (Fig. 2d) clavate to cylindrical, 11–23 × 2–3 µm, thin-walled, hyaline to pale brown, 0–1-septate. *Basal stroma* (Fig. 2f) 10–16 µm thick, consisting of several layers of brown, thick-walled cells, arranged in *textura angularis*, becoming colourless, thin-walled cells of *textura prismatica* towards the subhymenium. *Subhymenium* 19–27 µm thick, composed of several layers of hyaline, thin-walled cells of *textura angularis*. *Paraphyses* 1.5–2 µm, filiform, aseptate, unbranched, often curved, but not swollen at the apex, anastomosing at the base. *Asci* (81.5–)110–120(–129) × 10–14 µm (*x*¯ = 108 × 12 µm, n = 25), 8-spored, unitunicate, cylindrical-clavate, round to subtruncate at the apex, with a 38–49 µm long stalk, thin-walled, J-, apical ring, without circumapical thickening. *Ascospores* 26–32.5 × 2.5–4.5 µm (*x*¯ = 30.5 × 3.5 µm, n = 35, measured without the gelatinous sheath), multi-seriate and mostly arranged in the upper half of ascus, fusiform to slightly cylindrical, straight or lightly curved, apex rounded and tapering slightly to an acute base, aseptate, hyaline, guttulate, surrounded by a 0.5–1.5 µm thick gelatinous sheath (extending to 2.5 µm at the poles). *Asexual morph*: Not observed.

##### Material examined.

CHINA, Guizhou Province, Leishan County, dead stems of unidentified herbaceous plants, 2 November 2017, J.F. Zhang, LS-21 (GZAAS 19-1769, ***holotype***).

##### Notes.

Our phylogenetic analysis shows that *Hypoderma paralinderae* is placed in *Hypoderma* D clade (Fig. 1) and clustered with *H. cordylines*, *H. hederae* and *H. rubi*. Both *H. paralinderae* and *H. codylines* have similar sized asci (110–122.5 × 5.5–7 µm vs. 90–140 × 11–16 µm); however, they can be distinguished by the different shape and size of ascospores (fusiform to slightly cylindrical, 26–32.5 × 2.5–4.5 µm in *H. paralinderae* vs. elliptic, 14–21 × 4.5–6 µm in *H. cordylines*) (Johnston 1990). *Hypoderma paralinderae* shares similar-sized asci with *H. hederae*; however, it is differentiated from the latter by larger ascospores (26–32.5 × 2.5–4.5 µm vs. 18–22 × 3.5–4 µm) (Powell 1974). Moreover, *H. hederae* was described with oblong-cylindrical ascospores that are bluntly round on both ends; however, the ascospores in *H. paralinderae* are fusiform to cylindrical, but rounded at the apex and tapering slightly to an acute base (Powell 1974), while *H. paralinderae* differs from *H. rubi* by having obviously larger asci (110–122.5 × 5.5–7 µm vs. 60–100 × 10–12.5 µm) and ascospores (26–32.5 × 2.5–4.5 µm vs. 14–18 × 3.5–4.5 µm) (Hou et al. 2007). Besides, the recommendations of delineation taxa from Jeewon and Hyde (2016) are followed and comparisons of the ITS gene region between *H. paralinderae* and *H. cordylines* (ICMP 17344), as well as *H. paralinderae* and *H. rubi* (ICMP 17339) are processed. The results showed that there are 9/468 bp (1.9%) and 9/467 (1.9%) bp differences (including gaps) between them, respectively. According to the above evidence, *H. paralinderae* is introduced herein as new to science.

### *Terriera* B. Erikss., Symb. bot. upsal. 19(no. 4): 58 (1970)

*Terriera* was segregated from *Lophodermium* by Eriksson (1970) with *T. cladophila* as its type species. Johnston (2001) elucidated some distinctive morphological features (described as oblong to sublinear ascomata with single longitudinal opening slit, narrow-cylindrical asci and 1-septate ascospores that taper slightly at both ends and often becoming gently sigmoid on release and lacking a gelatinous sheath) for this genus and justified its monophyletic classification. There are 38 species accepted in *Terriera* (Index Fungorum 2020) and around half of these species were discovered recently from China (Chen et al. 2011, 2013; Yang et al. 2011; Zheng et al. 2011; Gao et al. 2012; Song et al. 2012; Zhou et al. 2012; Li et al. 2015a, b; Lu et al. 2015; Wu et al. 2015; Cai et al. 2020). Here, we introduce three novel species. These three species share morphological characters typical of *Terriera* and cluster together with existing *Terriera* species in LSU-ITS-mtSSU phylogenetic analyses. In addition, a synopsis for *Terriera* species is also provided and listed in Table 2.

**Table 2.** Synopsis of *Terriera* species. The new species described in this study are indicated in bold.

Species	Host	Appearance of apothecia	Asci	Ascospores	Origin	References
*Terriera aequabilis*	On dead leaves of *Photinia villosa*	Elliptical to sub-circular, straight or slightly curved to one side, ends rounded and opening by a single longitudinal split	75–105 × 4.5–5.5 µm	55–78 × 0.8–1 µm, filiform, aseptate, ends rounded, covered by a 0.3–0.5 µm wide gelatinous sheath	Jiangxi, China	Li et al. 2015b
*T. angularis*	On leaves of *Illicium simonsii*	Triangular to quadrangular, rarely elliptical and opening by 3–4 radial splits or a longitudinal split	105–130 × 5.5–6.5 µm	70–90 × 1–1.2 µm, filiform, aseptate, slightly tapering towards the round base, covered by a 0.8–1 µm wide gelatinous sheath	Hubei, China	Zhou et al. 2013
*T. arundinacea*	On decomposed leaves of *Bambusa* sp.	Oblong to sublinear and opening by a single longitudinal split	130–160 × 8–9 µm	90–100 × 2–2.5 µm, slightly tapering towards the base, lacking gelatinous sheath	Java, Indonesia	Johnston 2001
*T. asteliae*	On dead leaves of *Asterlia* sp.	Elliptical to oblong, ends rounded, opening by a single longitudinal slit	75–105 × 8–10.5 µm	45–70 × 2–2.5 µm, slightly tapering towards both ends and slightly constricted near the centre, aseptate or 1-septate, gently curved, lacking gelatinous sheath	Northland, New Zealand	Johnston 2001
*T. breve*	On dead leaves of *Carex*, *Unicinia* and *Gahnia* spp.	Oblong-elliptical, ends rounded, often sublinear, with a single longitudinal opening slit	110–135(–160) × 6–7 µm	(55–)60–75 × 1.5–2 µm, slightly tapering towards both ends, aseptate or 1-septate, gently curved or sigmoid, lacking gelatinous sheath	Campbell I, New Zealand	Johnston 2001
*T. camelliae*	On fallen leaves of *Camellia* sp.	Subcircular to irregular bleached spots, elliptical or occasionally 3-lobed and opening by a longitudinal split	85–120 × 5.5–6.5 µm	52–80 × 1–1.2 µm, filiform, aseptate, covered by a ca. 0.5 µm wide gelatinous sheath.	Fuzhou, China	Chen et al. 2011
*T. camelliicola*	On twigs of *Camellia sinensis*	Elliptical, occasionally fusing to form elongated elliptical, opening by a single longitudinal split	80–110 × 5–7 µm	50–70 × 1 µm, filiform, aseptate, covered by a 0.5 µm wide gelatinous sheath.	Assam, India	Minter and Sharma 1982
*T. cladophila*	On dead twigs of *Vaccinium myrtillus*	Elliptical, rounded at the ends, with a longitudinal opening split	75–100 × 5.5–8 µm	60–70 × 1 µm, filiform, aseptate, lacking gelatinous sheath	Norway	Terrier 1942; Eriksson 1970
*T. clithris*	On dead leaves of unidentified monocotyledon	Cylindrical to linear, with longitudinal opening slit	110–120 × 6.5–7.0 µm	60–80 × 1–1.5 µm, slightly tapering towards both ends, lacking gelatinous sheath	Rio Grande Do Sul, Brazil	Johnston 2001
*T. coacervata*	On leaves of *Lithocarpus cleistocarpus*	Elliptical, sometimes branching into lobed or polygonal shapes, opening by a longitudinal split or by more than 3 lobes	90–130 × 6.0–7.0 µm	60–110 × 1.5–1.8 µm, filiform, aseptate, covered by a 1.0–1.5 µm wide gelatinous sheath	Anhui, China	Zheng et al. 2012
*T. dracaenae*	On dead leaves or stems of *Dracaena* sp.	Oblong to oblong-elliptical, ends rounded, opening by a single longitudinal split	130–140 (–160) × 6–7 µm	100 × 2 µm, 1-septate, lacking gelatinous sheath	California, USA	Johnston 2001
*T. elliptica*	On living twigs of *Rhododendron* sp.	Elliptical, ends rounded to subacute, opening by a longitudinal split	135–175 × 7–9 μm	60–85 × 1.5–2 μm, filiform, slightly tapering towards both ends, aseptate, covered by a 1–1.5 μm wide gelatinous sheath	Yunnan, China	Zhang et al. 2015
*T. fici*	On dead leaves of *Ficus vasculosa*	Rounded or subrounded, with conspicuous edge and opening by a single longitudinal split	90–115 × 4–5.5 µm	65–80 × 0.8–1 µm, filiform, aseptate, rounded to obtuse at the apex, slightly tapering towards the rounded or subacute base, covered by a 0.5 µm wide gelatinous sheath	Hainan, China	Wu et al. 2016
*T. fuegiana*	On dead leaves of *Rostkovia grandiflora*	Oblong elliptical to broad-elliptical, ends rounded, opening by a single, longitudinal slit	75–95 × 7–10 μm	60–65 × 1.5–2.5 μm, slightly tapering towards both ends, 1-septate, lacking gelatinous sheath	Tierra del Fuego, Argentina	Johnston 2001
*T. fourcroyae*	On dead leaves of *Furcraea* sp.	Oblong-elliptical, ends rounded, with a single longitudinal opening slit	95–110 × 5–6.5 µm	60–70 × 1.5–2.5 μm, slightly tapering towards both ends, gently coiled or sigmoid, 1-septate, lacking gelatinous sheath.	Sri Lanka	Johnston 2001
*T. guizhouensis*	On dead leaves of *Eriobotrya japonica*	Elliptical, occasionally curved, opening by a longitudinal split	88–107 × 4–6 µm	50–80 × 1–1.2 µm, filiform, slightly tapering towards both ends, aseptate, pluriguttulate, covered by a thin gelatinous sheath	Guizhou, China	Cai et al. 2020
*T. houjiashanensis*	On dead leaves of *Ilex cornuta*	Elliptical, often curved, occasionally confluent, opening by a longitudinal split	103–128 × 4–6 µm	73–82 × 0.6–0.9 µm, filiform, slightly tapering towards both ends, aseptate, pluriguttulate, covered by an inconspicuous gelatinous sheath	Anhui, China	Cai et al. 2020
*T. huangshanensis*	On leaves of Eurya muricata var. huiana	Elliptical, fusiform or subfusiform, straight or curved (lunate), sometimes 3-lobed or triangular, ends rounded to subacute, opening by a single longitudinal split	100–120 × 5–7 µm	58–90 × 1.5–2 µm, filiform, slightly tapering towards the base, aseptate, covered by a 1–1.5 µm thick gelatinous sheath	Anhui, China	Yang et al. 2011
*T. ilicis*	On dead leaves of *Ilex pernyi*	Elliptical, occasionally curved, triangular or confluent, opening by a longitudinal split	117–139 × 4–7 µm	52–84 × ca. 1 µm, filiform, slightly tapering towards both ends, aseptate, pluriguttulate, covered by a thin gelatinous sheath	Hubei, China	Cai et al. 2020
*T. illiciicola*	On dead leaves of *Lithocarpus cleistocarpus*	Subcircular to broad-elliptical, opening by a longitudinal split	90–135 × 4.0–5.0 µm	65–95 × 1 µm, filiform, aseptate, covered by an inconspicuous gelatinous sheath	Anhui, China	Zheng et al. 2011
*T. intraepidermalis*	On fallen leaves of *Photinia prunifolia*	Widely elliptical, sometimes elliptical or subcircular, occasionally triangular, straight or curved to one side slightly, ends round to obtuse, opening by a single longitudinal split or by three radial splits	90–135 × 5.5–7.5 µm	70–105 × 1–1.5 µm, with upper end rounded to obtuse, slightly tapering towards the rounded base, covered by a 0.5 μm wide gelatinous sheath	Hunan, China	Lu et al. 2015
*T. javanica*	On dead leaves of *Elettaria* sp.	Oblong-elliptical to sublinear, ends acute, opening by a single longitudinal slit	85–95 × 5.5–7 µm	50–60 × 1.5 µm, but the detailed morphological characters were not seen	Java, Indonesia	Johnston 2001
***T. karsti***	**On dead branch of unidentified host**	**Elliptical or oblong-elliptical, ends slightly acute to obtuse, with a single longitudinal opening split**	**(103–)110–122.5 × 5.5–7 µm**	**55–66 × 1.5–2.0 µm, filiform, gradually tapering towards both ends, aseptate, lacking gelatinous sheath**	**Guihzou, China**	**In this study**
*T. latiascus*	On dead leaves of *Euterpe* and *Heliconia* spp.	Oblong-elliptical, with a single longitudinal opening slit	80–95 × 7–8.5 µm	40–50 × 2–2.5 µm, with 1(–3)-septate, slightly tapering to both ends	Amazonas, Brazil	Johnston 2001
*T. longissima*	On dead leaves of Bambusaceae sp.	Oblong to sublinear, ends rounded, opening by a single, longitudinal slit	175–210 × 6–6.5 µm	Approximately 120–130 µm long, but the detailed morphological characters were not seen	Potaro-Siparuni region VII, Guyana	Johnston 2001
*T. mangiferae*	On dead leaves of *Aucuba japonica* and *Mangifera indica*	Ellipsoidal, with a longitudinal opening split	80–90 × 5–6 µm	70–80 × 1 µm, filiform, lacking gelatinous sheath	Java, Indonesia	Koorders 1907; Li et al. 2014
***T. meitanensis***	**On dead culms of unidentified host**	**Elliptical to oblong-elliptical, ends slightly acute to obtuse, opening by a single longitudinal split**	**(98.5–)113–125.5(–131.5) × 6–7.5 µm**	**47–54.5 × 1.5–2.5 µm, filiform, gradually tapering towards both ends, aseptate, lacking gelatinous sheath**	**Guizhou, China**	**In this study**
*T. nematoidea*	On dead leaves of *Gahnia* sp.	Elliptical to sublinear, with a single longitudinal opening slit	70–80 × 5–6.5 µm	30–35 × 1 µm, slightly tapering towards both ends, gently curved or sigmoid, 1-septate, lacking gelatinous sheath	Northland, New Zealand	Johnston 2001
*T. nitens*	On leaves of *Cyclobalanopsis myrsinifolia*	Suborbicular or broadly elliptical, straight or slightly curved, opening by a single longitudinal split	95–150 × 1–1.2 µm	68–115 × 0.8–1.2 µm, filiform, aseptate, round at the apex, slightly tapering towards the acute base, covered by a thin gelatinous sheath	Anhui, China	Chen et al. 2013
*T. pandani*	On dead leaves of *Pandanus* sp.	Oblong to oblong-elliptical, ends rounded, opening by a single longitudinal slit	100–120 × 5–6 µm	50–70 × 1–1.5 µm, lacking gelatinous sheath	San Juan, Puerto Rico	Johnston 2001
*T. pandanicola*	On dead leaves of *Pandanus* sp.	Elliptical, with rounded to subacute ends, opening by a longitudinal split	50–66 × 4–5 µm	55–78 × 1–2 µm, filiform, slightly tapering towards both ends, aseptate, lacking gelatinous sheath	Prachuap Khiri Khan, Thailand	Tibpromma et al. 2018
*T. petrakii*	On fallen leaves of *Smilax bracteata*	Elongate-elliptical, strongly curved or triangular, often coalesced, opening by a longitudinal split	85–110 × 4–5 µm	(60–)70–85 × 0.8 µm, filiform, aseptate, covered by a thin gelatinous sheath	Yunnan, China	Song et al. 2012
*T. rotundata*	On fallen leaves of *Quercus* sp.	Elliptical, occasionally triangular, ends rounded, opening by a longitudinal split or occasionally by teeth	90–120 × 4–5.5 µm	70–90(–95) × 0.8–1 µm, filiform, aseptate, lacking gelatinous sheath	Yunnan, China	Song et al. 2012
*T. sacchari*	On dead leaves and leaf bases of *Saccharum officinarum*	Narrow-oblong to sublinear, with a single longitudinal opening split	90–100 × 5–7 µm	50–60 × 1.5 µm, lacking gelatinous sheath	Hawaii, USA	Johnston 2001
*T. samuelsii*	On dead leaves of unidentified monocotyledon	Oblong to sublinear, ends rounded, opening by a single longitudinal slit	125–140 × 7–8 µm	(65–)75–90 × 2 µm, slightly tapering towards both ends, 1-septate, lacking gelatinous sheath	Amazonas, Brazil	Johnston 2001; 2003
***T. sigmoideospora***	**On dead fallen leaves of unidentified host**	**Elliptical, ends rounded to subacute, opening by a single longitudinal split**	**(93.5–)102–121 × 5–6 μm**	**79–95 × 5–2 μm, filiform, slightly tapering towards both ends, aseptate, lacking gelatinous sheath**	**Guizhou, China**	**In this study**
*T. simplex*	On fallen leaves of *Trachelospermum jasminoide*s	Elliptical to ovate, ends obtuse, rounded or slightly acute, opening by a single longitudinal split which is sometimes branched in the triangular ascomata	72–95(–105) × 4.8–5.2 µm	(45–)56–82 × 1–1.2 µm, filiform, slightly tapering towards the rounded base, covered by a 0.8–1 µm wide gelatinous sheath	Anhui, China	Gao et al. 2012
*T. stevensii*	On dead leaves of *Vincentia* sp.	Oblong, ends rounded, opening by a single longitudinal slit	100–125 × 5–6 µm	60–80 × 1.5–2 µm, lacking gelatinous sheath	Hawaii, USA	Johnston 2001
*T. thailandica*	On dead branch of unidentified host	Elliptical, ends rounded to subacute, opening by a longitudinal split	80–105 × 3.4–6.6 µm	38–60 × 1–1.5 µm, filiform, slightly tapering towards both ends, aseptate, lacking gelatinous sheath	Chiang Rai, Thailand	Hyde et al. 2016
*T. transversa*	On dead leaves of *Pandanus* sp.	Elliptical or oblong-elliptical, ends slightly acute to obtuse, opening by a single longitudinal split	70–86 × 5–6 µm	45–68 × 1–1.2 µm, filiform, slightly tapering towards both ends, aseptate, covered by a 0.5 µm wide gelatinous sheath	Hainan, China	Li et al. 2015a

#### Terriera karsti

Taxon classificationFungiRhytismatalesRhytismataceae

J.F. Zhang & J.K. Liu sp. nov.

321F406B-EEE2-597C-BC79-35C57E8527D0

Index Fungorum number: IF556901

Facesoffungi Number No: FoF06799

Figure 3

##### Holotype.

MFLU 18-2288.

##### Etymology.

Refers to the karst landscape where the holotype was collected.

##### Description.

**Figure 3.**
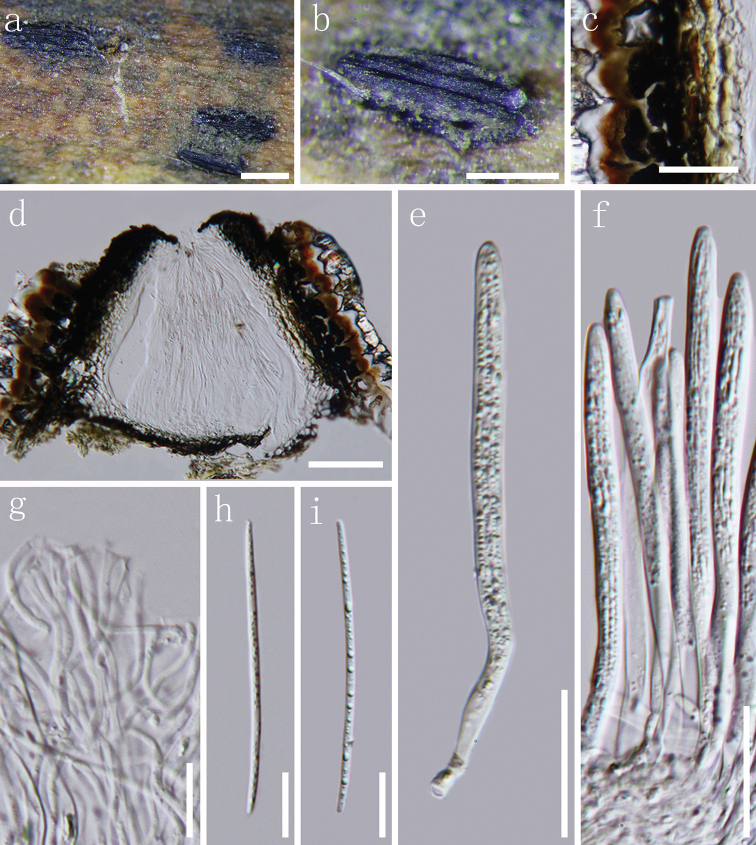
*Terriera karsti***a, b** apothecia observed under the dissecting microscope **c** detail of covering stroma in vertical section **d** vertical section through an apothecium **e, f** asci in various states of maturity **g** apices of paraphyses **h, i** ascospores. Note: **c–i** mounted in water. Scale bar: 1 mm (**a**), 500 µm (**b**), 20 µm (**c, e, f**), 100 µm (**d**), 10 µm (**g, i**).

*Apothecia* developing on dead branch, elliptical or oblong-elliptical in outline, ends slightly acute to obtuse. Apothecia surface black, matt or slightly glossy, moderately raising the substratum surface, opening by a single longitudinal split that extends to the ends of the apothecium (Fig. 3a, b). *Lips* absent. In median vertical section (Fig. 3d), apothecia deeply embedded in host tissue, with host cells becoming filled with fungal tissue as the apothecium develops. *Covering stroma* (Fig. 3c) 30–45 µm thick, composed of blackish-brown to black, thick-walled cells of *textura angularis* towards the exterior and several layers of pale to nearly hyaline, thin-walled cells towards the interior. Along the edge of the apothecial opening, there is a flattened, 12–20 µm thick extension adjacent to the covering stroma that is composed of strongly melanised tissue with no obvious cellular structure. *Basal stroma* 8–18 µm thick, dark brown or blackish-brown, composed of angular to globose, thick-walled cells, 2.5–4 µm diam. A triangular space between the covering stroma and basal stroma consists of thin-walled, nearly hyaline to grey-brown cells arranged in *textura prismatica*. *Paraphyses* 1–2 µm, filiform, hyaline, septate, gradually swollen or branching once at the apex, embedded in gelatinous sheaths. *Asci* (103–)110–122.5 × 5.5–7 µm (*x*¯ = 113 × 6 µm, n = 20), 8-spored, unitunicate, cylindrical, long stalk, thin-walled, apex truncate to somewhat round, J-, without circumapical thickening. *Ascospores* 55–66 × 1.5–2.0 µm (*x*¯ = 61 × 1.8 µm, n = 25), fascicle, but not coiled, filiform, gradually tapering toward the ends, hyaline, aseptate, smooth-walled, straight or slightly curved, lacking gelatinous sheath. *Asexual morph*: Not observed.

##### Culture characteristics.

Colonies on PDA reaching 51 mm after 14 days at 25 °C, irregular in shape, cottony with moderately dense, fluffy aerial mycelium. At first, white, becoming slightly greyish in the centre, reverse side bronze in the centre and pale towards the edge.

##### Material examined.

CHINA, Guizhou Province, Guiyang, Yunyan District, dead branch of unidentified ligneous plants, 6 May 2016, J.F. Zhang, SH-06 (MFLU 18-2288, ***holotype***); *ibid*. (GZAAS 19-1720, ***isotype***); ex-type living culture, GZCC 19-0047.

##### Notes.

In the present study (Fig. 1), *Terriera karsti* is phylogenetically close to *T. camelliicola* and *T. thailandica* with moderate support (MPBP 63% and BYPP 1.00). *Terriera karsti* is not significantly distinguished from *T. camelliicola*, based only on morphological characters as they share similar-sized asci (110–122.5 × 5.5–7 µm vs. 85–120 × 5.5–6.5 µm) and ascospores (55–66 × 1.5–2 µm vs. 50–70 × 1 µm) (Johnston 2001). However, the ascospores of *T. camelliicola* are covered by a 0.5 µm wide gelatinous sheath, while this is not observed in *T. karsti* (Sharma 1982). In order to clarify their affinity, the recommendations of species delineation from Jeewon and Hyde (2016) were followed and the comparison of each gene region between these two taxa is processed and showed that there are 9/840 bp (1%) and 10/694 bp (14.4%) differences in LSU and mtSSU regions, respectively, while *T. karsti* can be easily differentiated from *T. thailandica* by its larger asci (110–122.5 × 5.5–7 µm vs. 80–105 × 3.4–6.6 µm) and ascospores (55–66 × 1.5–2 µm vs. 38–60 × 1–1.5 µm) (Hyde et al. 2016). A comparison of the LSU gene region between these two taxa has also been processed and the result showed that there are 3/838 bp (base pair) differences. Based on phylogenetic analyses, coupled with morphological distinction, *Terriera karsti* is introduced herein as a new species.

#### Terriera meitanensis

Taxon classificationFungiRhytismatalesRhytismataceae

J.F. Zhang & Z.Y. Liu sp. nov.

7364F767-3A22-50BE-908E-A87521B1B7B4

Index Fungorum number: IF556900

Facesoffungi Number No: FoF06798

Figure 4

##### Holotype.

MFLU 18-2299.

##### Etymology.

Referring to the locality of the holotype, Meitan County, Guizhou Province, China.

##### Description.

**Figure 4.**
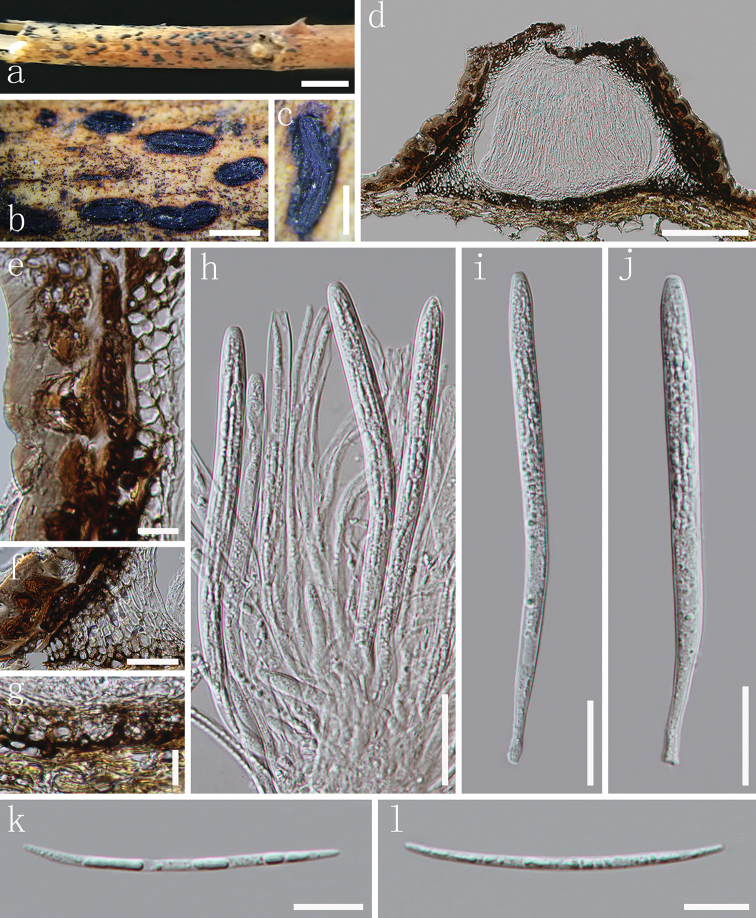
*Terriera meitanensis***a** habit of apothecia on substrate **b, c** apothecia observed under the dissecting microscope in face view **d** vertical section through an apothecium **e** covering stroma **f** triangular space in section between the covering stroma and basal stroma **g** basal stroma **h** paraphyses with anastomoses amongst asci in various states of maturity **i, j** immature asci **k, l** ascospores. Note: **d–l** mounted in water. Scale bar: 1 cm (**a**), 1 mm (**b**), 500 µm (**c**), 100 µm (**d**), 10 µm (**e, g, k, l**), 30 µm (**f**), 20 µm (**h–j**).

*Apothecia* developing on dead stems (Fig. 4a), semi-immersed to superficial, elliptical or oblong-elliptical, ends slightly acute to obtuse, surface black, matt, raising the substratum surface, opening by a single longitudinal split that extends nearly the entire length (Fig. 4b, c). In median vertical section (Fig. 4d), apothecia deeply embedded in host tissue, with host cells becoming filled with fungal tissue as the apothecium develops. *Covering stroma* (Fig. 4e) 33–42 µm thick, composed of blackish-brown, thick-walled cells that are fused with host tissue in the outermost layers, becoming pale pigmented or nearly colourless towards the hymenium, thin-walled cells, arranged in *textura angularis* or *textura globulosa.* Along the upper edge of the apothecial opening, there is a flattened, 19–34 µm thick extension adjacent to the covering stroma that is composed of strongly melanised tissue with no obvious cellular structure. *Basal stroma* (Fig. 4g) 8–18 µm thick, dark-brown or blackish-brown, composed of angular to globose, thick-walled cells, 2.5–4 µm diam. Where the covering stroma meets the basal stroma, there is a triangular-shaped, 35–60 µm thick, tissue composed of thin-walled, hyaline to pale brown cells forming a *textura prismatica* (Fig. 4f). *Subhymenium* 12–16 µm thick, consisting of hyaline *textura angularis* to *textura intricata*. *Paraphyses* 1–2 µm, filiform, hyaline, septate, gradually swollen or branching once at the apex, embedded in gelatinous matrix, anastomosing at the base. *Asci* (98.5–)113–125.5(–131.5) × 6–7.5 µm (*x*¯ = 117 × 6.5 µm, n = 20), 8-spored, unitunicate, cylindrical, somewhat long-stalked, thin-walled, apex generally truncate, J-, without circumapical thickening. *Ascospores* 47–54.5 × 1.5–2.5 µm (*x*¯ = 50.5 × 2 µm, n = 35), fascicle, filiform, gradually tapering towards the ends, hyaline, aseptate, smooth-walled, straight or slightly curved, lacking a gelatinous sheath. *Asexual morph*: Not observed.

##### Material examined.

CHINA, Guizhou Province, Zunyi, Meitan County, dead stems of unidentified host, 28 August 2017, J.F. Zhang, MT-1 (MFLU 18-2299, ***holotype***); *ibid*. (GZAAS 19-1731, ***isotype***).

##### Notes.

In our phylogenetic analysis (Fig. 1), *Terriera meitanensis* is placed in a robust clade with *T. camelliicola*, *T. elliptica*, *T. karsti* and *T. thailandica* by strong statistical support (MPBP 100% and BYPP 1.00). *Terriera meitanensis* has larger asci than *T. camelliicola* and *T. thailandica*, while the ascospores of *T. meitanensis* are smaller (Johnston 2001; Hyde et al. 2016). Both *T. meitanensis* and *T. karsti* share similar-sized asci, but *T. karsti* has larger ascospores (47–54.5 × 1.5–2.5 µm vs. 55–66 × 1.5–2.0 µm). *Terriera meitanensis* differs from *T. elliptica* by its obviously smaller asci (113–122.5 × 6–7.5 µm vs. 135–175 × 7–9 µm) and ascospores (47–54.5 × 1.5–2.5 µm vs. 60–85 × 1.5–2 µm) (Zhang et al. 2015). Moreover, the ascospores of *T. camelliicola* and *T. elliptica* are enveloped by a gelatinous sheath, respectively, while this is not observed in *T. meitanensis*. In addition, the comparison of the ITS gene region is processed between *T. meitanensis* and its closest species *T. elliptica*, based on the recommendations from Jeewon and Hyde (2016) and the results showed that there are 15/489 bp (3%) differences. Therefore, we introduce *T. meitanensis* herein as a new species, based on morphological and molecular evidence.

#### Terriera sigmoideospora

Taxon classificationFungiRhytismatalesRhytismataceae

J.F. Zhang & K.D. Hyde sp. nov.

19F42846-99D2-5FCD-B177-A58B33DAFFE3

Index Fungorum number: IF556902

Facesoffungi Number No: FoF06800

Figure 5

##### Holotype.

MFLU 18-2297.

##### Etymology.

Refers to its sigmoidal ascospores.

##### Description.

**Figure 5.**
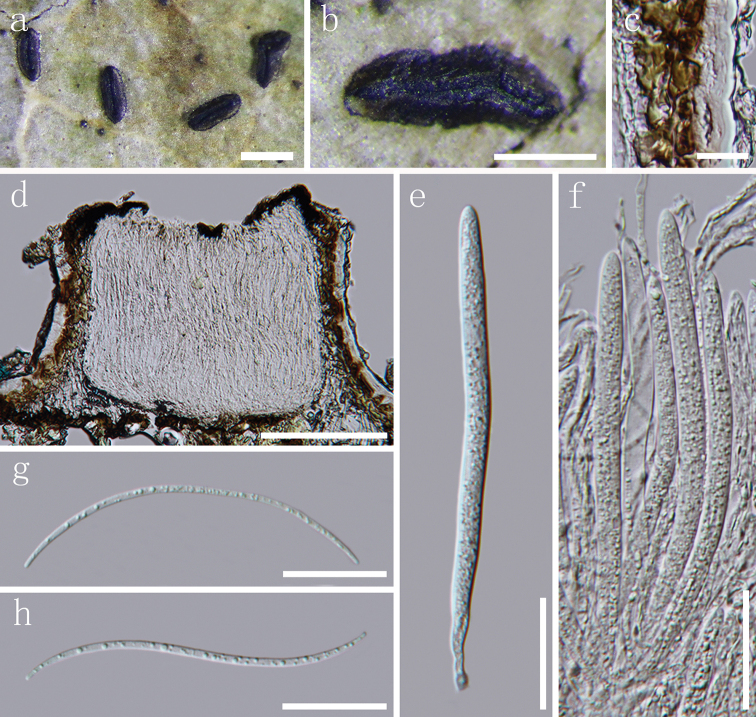
*Terriera sigmoideospora***a, b** apothecia observed under the dissecting microscope **c** section of covering stroma **d** median vertical section through an apothecium **e** immature ascus **f** paraphyses and asci in various states of maturity **g, h** ascospores. Note: **c–h** mounted in water. Scale bar: 1 mm (**a**), 500 µm (**b**), 100 µm (**c**), 20 µm (**d–h**).

*Apothecia* developing on fallen leaves, scattered, dark brown to black, matt, elliptical, sometimes 3-lobed or triangular, straight or slightly curved, ends rounded to subacute, strongly raising the surface of the substrate at maturity, opening by a single longitudinal split that extends almost the whole length of the apothecium (Fig. 5a, b). Immature apothecia appearing as a single dark brown protrusion, circular to slightly elongated. In median vertical section (Fig. 5d), apothecia 185–220 μm deep. *Covering stroma* (Fig. 5c) 20–25 μm thick near the centre of the apothecium, consisting of an outer layer of host cuticle, remains of epidermal and hypodermal cells filled with thick-walled, angular fungal cells and an inner layer of *textura angularis* to *textura globulosa* with 4–7 μm diam., dark brown, thick-walled cells, slightly thinner towards the edges, extending to the basal stroma, but conspicuously thicker towards the apothecial opening, with a 15–27 μm thick extension comprising highly melanised tissue with no obvious cellular structure. *Excipulum* moderately developed, closely adhering to the covering stroma and the extension, arising from the marginal paraphyses, becoming thinner towards the base. *Basal stroma* concave, 12–15 μm thick, composed of dark brown, thick-walled, angular cells. A triangular space between the covering stroma and basal stroma is composed of thin-walled, colourless cells that are vertically arranged in rows. *Subhymenium* 6–9 μm thick, flat, consisting of hyaline cells of *textura intricata*. *Paraphyses* filiform, hyaline, septate, gradually or suddenly swollen to 2.5 μm near the apex, covered by a thin gelatinous sheath, forming a 4–8 μm thick epithecium. *Asci* (93.5–)102–121 × 5–6 μm (*x*¯ = 108.5 × 5.5 µm, n = 20), 8-spored, unitunicate, cylindrical, apex tapering to round, thin-walled, J-, without circumapical thickening. *Ascospores* 79–95 × 1.5–2 μm (*x*¯ = 89.5 × 1.9 µm, n = 30), fascicle, filiform, sigmoid, tapering slightly towards the ends, hyaline, aseptate, guttulate, gelatinous sheath not observed. *Asexual morph*: Not observed.

##### Material examined.

CHINA, Guizhou Province, Guiyang, dead leaves of unidentified host, 5 October 2016, J.F. Zhang, GZ-28 (MFLU 18-2297, ***holotype***); *ibid*. (GZAAS 19-1729, ***isotype***).

##### Notes.

In the present phylogenetic analysis (Fig. 1), *Terriera sigmoideospora* is placed within *Terriera* and is related to *T. houjiazhuangensis* C.L. Hou & S.R. Hou by strong statistical support (MPBP 99% and BYPP 1.00). *Terriera sigmoideospora* shares similar-sized asci with *T. houjiazhuangensis* (102–121 × 5–6 μm vs. 103–128 × 4–6 μm), but has larger ascospores (79–95 × 1.5–2 μm vs. 73–82 × 0.6–0.9 μm) (Cai et al. 2020). Besides, the ascospores of *T. houjiazhuangensis* are enveloped by an inconspicuous gelatinous sheath, while this is not observed in *T. sigmoideospora*. In addition, the comparison of the ITS gene region between these two taxa has been processed and showed that there are 19/815 (2.3%) bp differences. *Terriera pandanicola* is sister to the above two taxa; however, it is significantly distinguished from *T. sigmoideospora* as its obviously smaller asci (50–66 × 4–5 μm vs. 102–121 × 5–6 μm) and ascospores (55–78 × 1–2 μm vs. 79–95 × 1.5–2 μm) (Tibpromma et al. 2018).

## Discussion

The diversity of microfungi in many parts of the world is understudied. This is evident from the numerous new species being described from Asia and South America (Hyde et al. 2018, 2019a, 2020). With this in mind, we are studying the fungi of the Karst regions in China and Thailand, where we are also finding numerous new species (Zhang et al. 2016, 2017a, b, 2018, 2019). Our study is contributing to the knowledge of fungal diversity in the region, where species may also have biotechnological potential (Hyde et al. 2019b). Additionally, as Rhytismataceae is a relatively poorly studied group, we report on one new species from *Hypoderma* and three new *Terriera* species, thereby illustrating the diversity and potential for new discoveries of these fungi in Asia.

*Hypoderma*, a large genus in Rhytismataceae, is a complicated group. There are only a few species in this genus with sequence data, but these have shown the group to be polyphyletic (Lantieri et al. 2011; Wang et al. 2013). This is also true of the phylogenies in this study (Fig. 1). *Hypoderma* is morphologically similar to *Lophodermium* and they mainly differ on the basis of ascospore shape as the former have elliptical to cylindrical-fusiform ascospores, while the latter has filiform ascospores (Powell 1974). However, there are no molecular studies that provide a natural classification for these two genera, even though more than 35 species have been synonymized under *Lophodermium* (Index Fungorum 2020). Fresh collections and molecular sequences are required to move toward a revision of these genera.

*Terriera* is one of the few genera in Rhytismataceae that can be considered a monophyletic group, based on distinctive morphology and phylogenetic characterisation (Zhang et al. 2015). Our molecular analyses corroborate this. However, there are only nine taxa with available sequences in GenBank and most of *Terriera* species were established, based only on morphological features (Yang et al. 2011; Gao et al. 2012; Song et al. 2012; Zhou et al. 2012; Chen et al. 2013; Li et al. 2015b; Lu et al. 2015; Zhang et al. 2015; Cai et al. 2020). In the latest study (Cai et al. 2020), *T. pandanicola* was distant from *Terriera* in ITS analysis, but included in this group on the basis of concatenated LSU-mtSSU sequence data. Cai et al. (2020) indicated that this taxon should be revised in a future study. Based on their suggestion, we checked the sequence data of *T. pandanicola* and found that the ITS sequence of this species is misidentified as it is not a related *Terriera* or even a Rhytismataceae species in BLASTn results. However, the newly generated available sequences (ITS and mtSSU) of *T. pandanicola* have been uploaded in GenBank and included in our phylogenetic analysis and the results indicated that it is a unique species in *Terriera* in the present study (Fig. 1).

## Supplementary Material

XML Treatment for Hypoderma paralinderae

XML Treatment for Terriera karsti

XML Treatment for Terriera meitanensis

XML Treatment for Terriera sigmoideospora

## Supplementary materials

Supplementary material 1 Dataset for molecular analyses Data type phylogenetic Explanation note The dataset of combined of LSU_ITS_mtSSU to build the phylogenetic tree. https://binary.pensoft.net/file/492867This dataset is made available under the Open Database License (http://opendatacommons.org/licenses/odbl/1.0/). The Open Database License (ODbL) is a license agreement intended to allow users to freely share, modify, and use this Dataset while maintaining this same freedom for others, provided that the original source and author(s) are credited.Jin-Feng Zhang, Jian-Kui Liu, Kevin D. Hyde, Anusha H. Ekanayaka, Zuo-Yi Liu
